# Blood Flow Restriction Training Using the Delfi System Is Associated With a Cellular Systemic Response

**DOI:** 10.1016/j.asmr.2020.09.009

**Published:** 2020-12-27

**Authors:** Mark C. Callanan, Hillary A. Plummer, Garrett L. Chapman, Tyler J. Opitz, Nicole K. Rendos, Adam W. Anz

**Affiliations:** a The Orthopedic Clinic, Shreveport, Louisiana, U.S.A.; b Andrews Research & Education Foundation, Gulf Breeze, Florida, U.S.A.; c Beaver Medical Group, Redlands, California, U.S.A.; d Andrews Institute for Orthopedics & Sports Medicine, Gulf Breeze, Florida, U.S.A.

## Abstract

**Purpose:**

To determine the effects of blood flow restriction (BFR) exercise on CD34^+^ cells, platelets, white blood cells, neutrophils, lymphocytes, lactate, and glucose.

**Methods:**

Healthy participants aged 20 to 39 years who were able to perform the exercise sessions were recruited. Participants underwent an experimental (EXP) occluded testing session and a control (CON) session using the Delfi Personalized Tourniquet System. Blood draws were performed prior to testing and immediately after the exercise session. Blood analysis consisted of a complete blood count as well as flow cytometry to measure peripheral CD34^+^ counts as a marker for hematopoietic progenitor cells.

**Results:**

Fourteen men (aged 30.8 ± 3.9 years) volunteered. There was a significant increase in average CD34^+^ counts immediately after the EXP session only (3.1 ± 1.2 cells ⋅ μL^–1^ vs 5.2 ± 2.9 cells ⋅ μL^–1^, *P* = .012). Platelet counts were significantly elevated after both sessions, with the average increase being higher after the EXP session (mean difference [MD], 34,200/μL; *P* < .002) than after the CON session (MD, 11,600/μL; *P* < .002). White blood cell counts significantly increased after both the EXP (8,400 ± 2,200/μL vs 6,300 ± 1,600/μL; *P* < .001) and CON (MD, 900/μL; *P* < .001) sessions. There was a significant increase from baseline to immediately after exercise in the average number of lymphocytes (MD, 6.3%; *P* < .001) and, conversely, a significant decrease in the average neutrophil count (MD, 6.5%; *P* < .001) in the EXP session only. Lactate levels significantly increased in the EXP (MD, 6.1 mmol ⋅ L^–1^; *P* = .001) and CON (MD, 3.6 mmol ⋅ L^–1^; *P* = .001) groups. No changes in glucose levels were observed.

**Conclusions:**

Exercise with BFR causes a significant post-exercise increase in peripheral hematopoietic progenitor cells and platelets, beyond that of standard resistance training.

**Clinical Relevance:**

BFR can be considered a way to manipulate point-of-care blood products such as platelet-rich plasma to increase product yield.

Blood flow restriction (BFR) therapy is becoming a part of orthopaedic rehabilitation, showing promise in muscle recovery as well as limb salvage after injury and orthopaedic surgery.^1^, ^2^, ^3^, ^4^ BFR is associated with functional, physiological, and cellular expression of genes related to muscle upregulation, similarly to heavy-load strength training.^5^^,^^6^ Low-load BFR can result in increases in muscular size and strength, even in proximal muscle groups that are not directly occluded. The same ability to achieve increases in proximal muscle size and strength has not been shown in matched controls undergoing traditional training methods.^7^ Even in well-trained athletes, BFR has been shown to increase strength and hypertrophy using submaximal loads that otherwise would not have the same response in a control group.^8^, ^9^, ^10^, ^11^ BFR has been studied in the postoperative care of patients after knee arthroscopy and anterior cruciate ligament reconstruction, with improved strength and patient-reported outcomes compared with conventional therapy, as well as diminishment in the degree of disuse atrophy.^3^^,^^12^ BFR is a viable option to improve muscle strength in patients unable to perform high-intensity exercise who have ultimately not improved with traditional therapy.^2^^,^^4^^,^^11^

Although the mechanism of action of increased muscle strength and hypertrophy from BFR is not completely understood, lactate and growth hormone levels increase from 0 to 40 minutes after BFR.^13^, ^14^, ^15^, ^16^, ^17^ Exercise with BFR is associated with low skeletal muscle tissue oxygenation saturation levels (<10%) as measured by near-infrared spectroscopy, representing severe hypoxia in the working tissue.^18^ The metabolic overload from the accumulation of hydrogen and lactate, in combination with the hypoxia, may activate IL-6, macrophages, and neutrophils, leading to an overall anabolic environment without the mechanical muscle damage that occurs with high-intensity training.^19^ Increased signaling and proliferation of local myogenic stem cells in post-therapy muscle biopsy samples have been observed after BFR therapy.^20^, ^21^, ^22^ BFR has also been shown to induce a local angiogenic response through upregulation of vascular endothelial growth factor, another proposed mechanism for the noted efficacy of BFR therapy.^23^

Another rapidly growing area of interest in orthopaedic surgery and recovery science is the clinical use of stem cells. Adult stem cells have the ability to monitor their local and systemic environment for stimuli, mobilize locally and/or systemically in the setting of an environmental insult such as exercise, interact with their surrounding environment through paracrine effects, and differentiate to end-stage cells if necessary.^24^, ^25^, ^26^ In rat models, heat, hypoxia, and cold can stimulate stem cells to mobilize, with hypoxia-induced factors being upregulated as a key factor in peripheral migration of mesenchymal stem cells.^27^ An increase in the peripheral mobilization of platelets as well as hematopoietic stem cells after vigorous exercise in humans has also been observed.^25^^,^^26^^,^^28^^,^^29^ Exercise using BFR may be a less invasive method to mobilize stem cells to optimize the physiology of recovering orthopaedic patients, as well as to manipulate point-of-care blood and bone marrow products in orthopaedics.^28^

Despite the previously studied mechanisms of efficacy for BFR therapy, the degree of mobilization of the cellular components of blood including hematopoietic progenitor cells (HPCs) to the peripheral circulation after exercise with BFR is unclear. The purpose of this study was to determine the effects of BFR exercise on CD34^+^ cells, platelets, white blood cells (WBCs), neutrophils, lymphocytes, lactate, and glucose. It was hypothesized that BFR training would stimulate a systemic cellular response to increase CD34^+^ cells, platelets, WBCs, neutrophils, lymphocytes, lactate, and glucose.

## Methods

A randomized crossover-design study was performed with the Delfi PTS Personalized Tourniquet System (Owens Recovery Science, San Antonio, TX). A complete blood count (CBC) with WBC differential, flow cytometry to quantify the number of CD34^+^ HPCs, and blood lactate and glucose levels were measured prior to the exercise protocols (PRE) and at various time points after the exercise protocols.

Healthy adults aged 20 to 39 years were recruited to participate in this study. Participants were excluded if they had a history of uncontrolled hypertension, diabetes, autoimmune disorders, blood disorders, disorders requiring immunosuppression, or cancer; an ongoing infectious disease; use of steroids; or significant cardiovascular, renal, hepatic, or pulmonary disease. Furthermore, participants were excluded if they had a history of an orthopaedic injury within the past 6 months. All participants had to be medically fit to perform 20 minutes of intense exercise.

All procedures were approved by the Baptist Hospital-Pensacola institutional review board. Prior to data collection, all testing procedures, risks, and benefits of the specific study were explained to each participant and written informed consent was obtained. Each participant underwent a standard physical examination, including the completion of a medical history and assessment of activity level with the Tegner Activity Level scale. Once all screening processes were passed, the participants were enrolled for a testing appointment. Participants were asked to refrain from strenuous exercise for 24 hours and from alcohol and caffeine for 12 hours prior to each testing session.

An a priori power analysis (G∗Power, version 3.1.9.3) revealed that a sample size of 10 participants was necessary to detect large effects (200%) with a power of 0.9 and α of .05. Sufficient power has been confirmed in previous mobilization studies.^10^ The sample size of our study was increased to 14 to account for potential participant withdrawal. Fourteen participants completed the study. Participant characteristics are provided in Table 1.

**Table 1:** Demographic Characteristics of Participants Undergoing Exercise with Blood Flow Restriction Using the Delfi System

Characteristic	Data
Age, yr	30.8 ± 3.7
Height, m	1.8 ± 0.07
Weight, kg	89.6 ± 16.5
Tegner score	5.5 ± 1.1

NOTE. Data are presented as mean ± standard deviation.

Participants rested in the sitting position for 15 minutes prior to each testing session. A 6-mL volume of venous blood was drawn from an antecubital vein into two 3-mL blood collection tubes (Vacuette [454246]; Greiner Bio-One, Monroe, NC) before (PRE) and at various time points after the testing protocol. Three milliliters of whole blood was used to obtain a CBC with WBC differential using a Sysmex automated hematology analyzer (Sysmex America, Lincolnshire, IL). Flow cytometry (Cytomics FC500 Flow Cytometer; Beckman Coulter Life Sciences, Indianapolis, IN) was used to quantify the number of CD34^+^ HPCs present in the peripheral blood.

Finger-stick capillary samples were used to evaluate blood lactate and glucose levels. A Lactate Plus portable lactate analyzer (Nova Biomedical, Waltham, MA) and Contour Next blood glucose meter (Ascensia Diabetes Care US, Parsippany, NJ) were used to measure blood lactate and blood glucose levels, respectively. The fingers were cleaned with an alcohol swab; then, a single-use lancet was used to puncture the finger for blood testing. Both sides of the puncture site were pressed gently as needed to develop a drop of blood. The first drop of blood was wiped off using a sterile cotton swab to avoid contamination with interstitial fluid. When the second drop of blood had developed, the test strip for each meter was touched to the blood drop until the unit meter beeped. Different testing fingers were used for each finger stick. All samples were handled under universal precautions.

Each participant attended 3 testing sessions: a familiarization session followed by 2 testing sessions. The familiarization session occurred between 3 days and up to 2 weeks before the first experimental testing session. The 2 experimental testing sessions occurred within a minimum of 48 hours and a maximum of 2 weeks between sessions. Each participant completed an experimental (EXP) testing session using the Delfi system and a control (CON) testing session using the same exercise protocol without the Delfi system. The order for EXP and CON sessions was randomized among participants.

Height, weight, and blood pressure were obtained on presentation for the familiarization session. All participants were then introduced to each of the exercise machines and proper use was demonstrated. The exercise machines used during testing included a seated leg extension machine, a semi-reclined leg press machine, and a seated hamstring curl machine.

The 1-repetition maximum (1-RM) for each exercise was determined during the first familiarization session using a standard algorithm. The resistance of each exercise machine was subsequently increased until the participant was only able to perform a single repetition to determine the participant’s maximum. This process was repeated for each exercise (seated leg extension, semi-reclined leg press, and seated hamstring curl) until all 1-RM values were determined.

Participants completed 2 testing sessions separated by a minimum of 48 hours and within 2 weeks of the familiarization session in a randomized order. The standardized blood draw protocol was used to obtain PRE blood samples. Participants completed the EXP and CON sessions under the supervision of an investigator (T.J.O.) trained in use of the Delfi system. During the EXP session, bilateral proximal thigh tourniquets were applied and inflated to a pressure of 80% of occlusive pressure as determined by the automated tourniquets. During the CON session, participants completed the same exercise protocol without the use of the Delfi system. Each participant then completed the 3 exercises (seated leg extension, prone hamstring curl, and semi-reclined leg press) with a format of 1 set each of 30, 15, 15, and 15 repetitions per exercise with 30 seconds of rest between sets while using the Delfi system at 80% limb occlusion pressure. The resistance for each exercise was set at 30% of the predetermined 1-RM. The tourniquets were deflated between exercises for 1 minute after the 4 sets had been completed. The tourniquets were reinflated at 80% occlusion prior to beginning each subsequent exercise until all exercises were completed. The exercise bout of a specific exercise was terminated prematurely if participants reached failure and were unable to complete 3 repetitions in a row; participants were then instructed to complete the subsequent exercise set.

Post-exercise blood samples were collected immediately after exercise (T0) and again at the 20-minute (T20), 40-minute (T40), and 60-minute (T60) time points from a peripheral intravenous line that was placed immediately after the training session. Finger-stick blood lactate and blood glucose measurements were also taken at T0 and at 10-minute intervals for 60 minutes after the training session (10 minutes [T10], T20, 30 minutes [T30], T40, 50 minutes [T50], and T60). The remaining testing session (EXP or CON) was repeated on a second testing day using the same protocol. A baseline blood sample was also taken on the second day of testing.

Repeated-measures analyses of variance (ANOVAs) were used to detect differences between the EXP and CON sessions and among time points for each outcome variable. Dependent variables included the WBC count (per microliter), platelet count (per microliter), percentages of neutrophils and lymphocytes in the WBC differential, CD34^+^ count (cells per microliter), blood lactate level (millimoles per liter), and blood glucose level (milligrams per deciliter). Statistical significance was set a priori at *P* < .05, and all analyses were performed using IBM SPSS Statistics software (version 24.0; IBM, Armonk, NY).

Separate 2 (session) × 5 (time) repeated-measures ANOVAs were used to detect differences between the EXP and CON sessions among the 5 time points (PRE, T0, T20, T40, and T60) for WBC count, platelet count, percentage of neutrophils, percentage of lymphocytes, and CD34^+^ count. Additional 2 (session) × 8 (time) repeated-measures ANOVAs were used to detect differences between the EXP and CON sessions among the 8 time points (PRE, T0, T10, T20, T30, T40, T50, and T60) for lactate and glucose levels. If the Mauchly test of sphericity was statistically significant (*P* < .05), a Huynh-Feldt adjustment was used to correct for the violation of sphericity. Simple effects were used to investigate a 2-way interaction, and pair-wise comparisons with a Bonferroni correction for multiple comparisons were used with a significant main effect of time.

## Results

Fourteen healthy men (age, 30.8 ± 3.9 years; height, 179.7 ± 7.3 cm; and weight, 89.6 ± 17.1 kg) volunteered to participate. The mean Tegner Activity Level score for the participants was 5.5 ± 1.1 (Table 1). There was a significant increase in average CD34^+^ counts immediately after the EXP session at T0 only (3.1 cells ⋅ μL^–1^ vs 5.2 cells ⋅ μL^–1^; PRE range, 1.5-9 cells ⋅ μL^–1^; T0 range, 1.5-12.5 cells ⋅ μL^–1^; *P* = .012). These values normalized by 20 minutes and beyond after the exercise session (Table 2). One participant’s CD34^+^ data for the EXP session were removed because of outliers greater than 3 standard deviations above the mean.

**Table 2:** Complete Blood Count With Differential and Flow Cytometry Results

Variable	PRE	T0	T20	T40	T60
WBC count					
Experimental, 1,000 ⋅ μL^–1^					
Participant 1	4.5	5.8	4.3	3.9	4.8
Participant 2	5.2	8.4	5.6	5	4.7
Participant 3	7.3	8	7.6	6.5	6.4
Participant 4	3	4.4	3.2	3.1	3.3
Participant 5	6.7	9.4	6.2	5.9	5.7
Participant 6	8.1	10.3	7.6	7.3	7
Participant 7	7.3	11.7	8.6	7.1	7.2
Participant 8	6.2	9.2	6.7	5.7	5.7
Participant 9	6.5	8	5.9	5.2	5.4
Participant 10	3.6	5.3	3.8	3.3	3.2
Participant 11	7.6	9.4	7.7	7.3	7.3
Participant 12	4.7	6.1	4.5	4.1	4
Participant 13	7	10.7	7.5	6.6	6.5
Participant 14	5.7	9	6.8	5.1	4.8
Mean ± SD	6.0 ± 1.6	8.3 ± 2.2∗†	6.3 ± 1.7	5.6 ± 1.5‡	5.6 ± 1.5‡
95% CI	5.2-7.0	7.2-9.7	5.4-7.2	4.8-6.4	4.8-6.4
Range	3-8.1	4.4-11.7	3.2-8.6	3.1-7.3	3.2-7.3
Δ from PRE for experimental, %		37.7	3.3	–8.2	–8.2
Control, 1,000 ⋅ μL^–1^					
Participant 1	7.9	8.8	8.1	8.2	7.2
Participant 2	5.6	6.7	5.9	5.7	5.7
Participant 3	6.7	7.4	6.2	6	5.9
Participant 4	4.3	4	3.5	3.5	3.5
Participant 5	7.1	7.6	7.2	7.3	7.3
Participant 6	7.7	8.6	7.3	6.8	7.4
Participant 7	9.2	11.5	9.5	9	9.1
Participant 8	6	5.9	5	5.1	5.4
Participant 9	6.8	7.1	6.2	6.2	6.6
Participant 10	5.7	6.9	5.9	5.4	5.2
Participant 11	7.8	9.1	7.3	7.2	6.9
Participant 12	3.8	4.4	4	4.4	4.5
Participant 13	8.4	9.1	7.7	7.3	7.2
Participant 14	5.4	7.5	6.4	6.3	6.3
Mean ± SD	6.6 ± 1.5	7.5 ± 1.9∗	6.4 ± 1.5	6.3 ± 1.4	6.2 ± 1.4‡
95% CI	5.8-7.5	6.5-8.6	5.6-7.3	5.5-7.1	5.5-7.0
Range	3.8-9.2	4-11.5	3.5-9.5	3.5-9	3.5-9.1
Δ from PRE for control, %		13.6	–3.0	–4.5	–6.1
Platelets					
Experimental, 1,000 ⋅ μL^–1^					
Participant 1	250	288	254	247	260
Participant 2	255	306	271	270	268
Participant 3	203	153	203	190	197
Participant 4	182	202	172	165	172
Participant 5	254	294	251	232	238
Participant 6	227	265	238	237	236
Participant 7	364	439	388	350	354
Participant 8	227	289	230	222	229
Participant 9	208	246	195	196	193
Participant 10	187	216	197	181	173
Participant 11	197	225	199	194	184
Participant 12	169	154	132	141	63
Participant 13	245	325	275	254	237
Participant 14	257	299	260	238	230
Mean ± SD	230.4 ± 48.6	264.6 ± 72.2∗	234.8 ± 58.6	224.3 ± 50.3‡	218.8 ± 62.9†
95% CI	206.1-258.7	226.6-306.6	202.4-267.2	196.5-252.2	184.0-253.6
Range	169-364	153-439	132-388	141-350	63-354
Δ from PRE for experimental, %		14.7	1.03	–3.5	–13.6
Control, 1,000 ⋅ μL^–1^					
Participant 1	215	215	293	290	307
Participant 2	279	244	283	281	273
Participant 3	173	174	167	165	164
Participant 4	183	182	168	167	174
Participant 5	255	265	259	257	252
Participant 6	223	251	231	230	230
Participant 7	428	458	419	397	412
Participant 8	227	245	230	223	247
Participant 9	203	198	201	206	208
Participant 10	209	234	208	201	198
Participant 11	264	289	267	254	249
Participant 12	133	152	152	148	160
Participant 13	267	267	271	259	254
Participant 14	224	251	236	221	228
Mean ± SD	235.9 ± 66.1	247.5 ± 71.2∗	242.5 ± 71.2	236.7 ± 61.2	239.1 ± 62.8
95% CI	199.2-272.5	208.1-286.9	206.3-278.7	202.8-270.6	204.3-273.9
Range	133-428	152-458	152-419	201-397	160-412
Δ from PRE for control, %		4.9	2.3	0.3	1.4
Neutrophils					
Experimental, %					
Participant 1	51.1	45.5	52.2	55.3	63.4
Participant 2	51.5	36.8	49.5	49.7	52.4
Participant 3	66.6	64.6	66.9	68.5	68.3
Participant 4	46.1	44.6	50	49.9	50.9
Participant 5	44.5	41.2	47.7	49.7	52.5
Participant 6	47.3	39.6	46.6	48.8	50.5
Participant 7	54.9	44.6	48.4	54	58.9
Participant 8	49.2	43.6	45.1	46	48
Participant 9	46	43.2	48.4	53.2	52.9
Participant 10	65.5	53.7	61.5	65.7	66.5
Participant 11	55.4	53.1	56.1	59.3	60.7
Participant 12	61.7	53.9	63.1	62.5	60.5
Participant 13	50.8	43.1	49.3	52.7	56.7
Participant 14	46.6	36.2	40.6	45.8	50.1
Mean ± SD	52.7 ± 7.3	46.3 ± 7.6‡	52.0 ± 7.2	54.6 ± 6.9∗	56.8 ± 6.3∗
95% CI	48.9-56.7	42.1-50.6	48.0-56.0	50.7-58.4	53.3-60.3
Range	46.6-66.6	36.2-64.6	40.6-66.9	45.8-68.5	48-68.3
Δ from PRE for experimental, %		–12.3	–1.5	3.4	7.6
Control, %					
Participant 1	60.4	58.5	58.7	59.2	61.2
Participant 2	55.2	49.1	53	55.2	53.1
Participant 3	56.4	56.3	57.4	56.7	56.9
Participant 4	48.7	53.3	54.9	56.7	53
Participant 5	48.4	48.2	47.3	47.7	47.2
Participant 6	44.1	42.4	46.4	45.7	44.1
Participant 7	60.3	55.3	56.7	57.3	57.1
Participant 8	51.6	53.4	56.2	58.3	60.2
Participant 9	48.6	47.9	49.1	49.3	51.1
Participant 10	63.4	59.2	63.7	62.7	62.1
Participant 11	55.3	53.8	55.9	56.8	56.3
Participant 12	52.6	55.1	60.6	59.3	60
Participant 13	48	46.8	51.2	51.4	52
Participant 14	62.7	56.9	65.3	66.4	65.9
Mean ± SD	52.7 ± 7.6	51.5 ± 6.5	54.6 ± 6.5	55.0 ± 6.5∗	54.9 ± 6.6∗
95% CI	48.5-56.9	47.9-55.0	51.0-58.2	51.4-58.6	51.3-58.6
Range	44.1-63.4	42.4-59.2	46.4-65.3	45.7-66.4	44.1-65.9
Δ from PRE for control, %		–2.3	3.6	4.4	7.8
Lymphocytes					
Experimental, %					
Participant 1	34.8	40.1	33.7	30.2	24.1
Participant 2	35.8	52.2	39.3	38.5	35.5
Participant 3	23.3	25.5	23.6	22.6	23
Participant 4	42.6	44.4	40.3	40.2	39
Participant 5	37.8	41.4	34.8	31.9	30.3
Participant 6	37.5	45.8	39.2	36.9	35.1
Participant 7	30.4	40	36.3	30.9	26.8
Participant 8	34.5	40.2	37.8	37.7	35.7
Participant 9	42	45	39.9	35.8	35.3
Participant 10	17	26.5	19.9	17.2	16.6
Participant 11	31.8	34.9	32.2	29.6	27.2
Participant 12	31.2	37	29.1	30	30.1
Participant 13	41.1	47.8	40.1	37.7	34.3
Participant 14	40.7	50.5	46.6	41.4	37.1
Mean ± SD	34.3 ± 7.3	40.4 ± 7.8∗	34.9 ± 6.9	32.6 ± 6.7	30.4 ± 6.3‡
95% CI	30.2-38.1	36.0-44.7	31.1-38.8	28.9-36.3	26.9-33.9
Range	17-42.6	25.5-52.2	19.9-46.6	17.2-41.4	16.6-37.1
Δ from PRE for experimental, %		18.5	2.3	–4.4	–10.9
Control, %					
Participant 1	26.6	29	29.2	28.1	26.9
Participant 2	33.3	39.7	35.7	33.7	36.6
Participant 3	31.1	31.9	30.8	31.3	32.8
Participant 4	40	36.4	34.5	32.9	36.2
Participant 5	35	36.1	35.8	35.8	35.6
Participant 6	43.6	45.7	41.7	41.6	43.8
Participant 7	29.9	34.6	33.6	32.6	33.5
Participant 8	33.4	32.3	29.9	27.3	26.1
Participant 9	40.8	40.6	39.8	39.8	38.2
Participant 10	24.7	28.5	23.8	23.6	24
Participant 11	31.7	32.2	30.9	29.9	30.1
Participant 12	40	36.6	31	30.8	31.3
Participant 13	40	41.7	38.4	37.5	38
Participant 14	28.6	32.4	24.9	23.5	24.6
Mean ± SD	35.1 ± 6.7	36.4 ± 5.7	33.4 ± 5.5	32.6 ± 5.7‡	33.1 ± 5.9
95% CI	31.4-38.8	33.2-39.5	30.4-36.5	29.4-35.8	29.9-36.4
Range	24.7-43.6	28.5-45.7	24.9-41.7	23.5-39.8	24.6-38.2
Δ from PRE for control, %		3.7	–4.8	–7.1	–5.7
CD34^+^					
Experimental, cells ⋅ μL^–1^					
Participant 1	5.5	4	2.5	4.5	7
Participant 2	3	3.5	2.5	2.5	3.5
Participant 3§	—	—	—	—	—
Participant 4	1.5	2	3	1.5	1.5
Participant 5	4.5	7	4	4	4.5
Participant 6	4	6.5	4.5	4	5
Participant 7	3.5	9.5	4.5	4.5	4
Participant 8	3	4.5	2.5	2.5	2
Participant 9	3.5	4	3.5	2.5	2
Participant 10	1.5	1.5	1.5	1.5	1
Participant 11	9	12.5	9	7	6
Participant 12	2	11.5	4.5	3	4
Participant 13	2.5	5	2	2.5	2
Participant 14	2.5	2.5	1.5	1	1
Mean ± SD	3.1 ± 1.2	5.2 ± 2.9∗	3.1 ± 1.1	2.9 ± 1.2	3.2 ± 1.8
95% CI	2.5-3.8	3.8-6.7	2.6-3.7	2.3-3.5	2.3-4.1
Range	1.5-9	1.5-12.5	1.5-9	1-7	1-7
Δ from PRE for experimental, %		60	0	–7	3
Control, cells ⋅ μL^–1^					
Participant 1	3.5	11.5	4.5	8	7.5
Participant 2	3.5	4	3	3.5	3
Participant 3	8	7.5	8	6.5	6
Participant 4	2	2	1	1.5	1
Participant 5	5.5	4	2	3.5	4.5
Participant 6	5	6	3	3.5	1.5
Participant 7	5	5.5	5.5	4.5	5.5
Participant 8	5	3.5	3	3.5	2.5
Participant 9	2	3	1.5	2	2
Participant 10	3.5	6	3.5	4.5	3
Participant 11	7	5.5	6	7	6
Participant 12	9	3.5	5	10	3
Participant 13	3.5	3	2.5	2	2
Participant 14	1.5	2	1	1.5	1.5
Mean ± SD	4.6 ± 2.2	4.8 ± 2.4	3.5 ± 2.0	2.6 ± 1.8	3.5 ± 2.0
95% CI	3.4-5.8	3.5-6.8	2.4-4.6	1.7-3.6	2.4-4.6
Range	1.5-9	2-11.5	1-6	1.5-10	1-7.5
Δ from PRE for control, %		4.3	–23.9	–43.5	–23.9

CI, confidence interval; PRE, prior to exercise protocol; SD, standard deviation; T0, immediately after exercise; T20, 20 minutes after exercise; T40, 40 minutes after exercise; T60, 60 minutes after exercise; WBC, white blood cell.

§ CD34^+^ data were removed because of outliers greater than 3 SDs from the mean.

∗ Significant increase from PRE.

† Significant session × time interaction at specified time point.

‡ Significant decrease from PRE.

There was a significant increase in platelet counts immediately after the exercise session (T0) for both the EXP (232,400/μL vs 266,600/μL; PRE range, 169,000-364,000/μL; T0 range, 153,000-439,000/μL; *P* < .002) and CON (235,900/μL vs 247,500/μL; PRE range, 133,000-428,000/μL; T0 range, 152,000-458,000/μL; *P* < .002) testing sessions. The average increase was higher after the EXP session versus the CON session (mean difference [MD], 8,000/μL). These values normalized at T20 after exercise for both the EXP and CON testing sessions. After the EXP session only, a significant decrease in average platelet values was observed from baseline to T40 after the exercise session (232,400/μL vs 224,300/μL; T0 range, 141,000-350,000/μL; *P* < .01), which again normalized by T60 after the session (Table 2).

There was a significant increase in the average WBC counts from baseline to T0 after both the EXP (8,400/μL vs 6,300/μL; PRE range, 3,000-8,100/μL; T0 range, 4,400-11,700/μL; *P* < .001) and CON (PRE range, 3,000-9,200/μL; T0 range, 4,000-11,500/μL; *P* < .001) sessions (Table 2). This increase in WBC counts was higher after the EXP session versus the CON session (MD, 900/μL; *P* < .001) (Table 2). There was a significant increase from baseline to T0 in the average number of lymphocytes (34.1% vs 40.4%; PRE range, 17%-42.6%; T0 range, 25.5%-52.2%; *P* < .001) and, conversely, a significant decrease in the average neutrophil count (52.8% vs 46.3%; PRE range, 46.6%-66.6%; T0 range, 36.2%-64.6%; *P* < .001) in the EXP session only. These findings initially normalized by T20, but then a significant decrease in average lymphocyte count from baseline was observed at T60 (34.1% vs 30.4%; T60 range, 16.6%-37.1%; *P* < .001). A significant increase in average neutrophil count at both T40 (52.8% vs 54.6%; T40 range, 45.8%-68.5%; *P* < .001) and T60 (52.8% vs 56.8%; T60 range, 48%-68.3%; *P* < .001) was also observed after the EXP session.

There were no significant changes from baseline to post-workout glucose levels after either training session at any time point (Table 3). There was a significant increase in lactate levels immediately after the workout for both the EXP (MD, 6.1 mmol ⋅ L^–1^; *P* = .001) and CON (MD, 3.6 mmol ⋅ L^–1^; *P* = .001) training sessions, which remained significantly elevated until T40, when the values normalized. The noted average increase in lactate levels was higher after the EXP training session at all time points up to T40 (Table 3).

**Table 3:** Blood Lactate and Glucose Levels Results

Variable	PRE	T0	T10	T20	T30	T40	T50	T60
Lactate, mmol ⋅ L^–1^								
Experimental								
Participant 1	3.1	4.7	4.7	2.3	4.2	3.5	1.4	1.7
Participant 2	3.2	7.6	6	4.2	4.1	3.8	2.5	3.1
Participant 3	1.8	5.4	5.2	4.5	2.4	2.5	1.7	2.1
Participant 4	1.1	4.7	3.4	2.3	2.3	2.3	1.2	1.1
Participant 5	1.3	6.2	4.5	3.4	2.3	2.4	1.7	2.2
Participant 6	1.8	5.5	5.6	3.1	3.2	2.8	1.3	1.1
Participant 7	2	11.5	12	10	8.1	6	4.4	4.9
Participant 8	1.9	6.3	5.4	4.4	3.2	2.2	2.1	1.3
Participant 9	0.5	8	7.9	7.2	6.3	6.2	5.8	3.1
Participant 10	1.6	10.7	11	8.3	5.4	5.3	3.7	2.6
Participant 11	2.5	5.8	5.1	5.9	2.8	2.4	2.8	2.1
Participant 12	1.6	8	5.8	5.7	4.6	2.6	2.4	2.6
Participant 13	1.7	9.8	10.3	8.7	4.4	3.9	4.1	2.5
Participant 14	1.7	13.8	13.1	11.2	9.5	6.6	6.1	4.2
Mean ± SD	1.8 ± 0.7	7.87 ± 2.8∗†	7.1 ± 3.0∗†	6.0 ± 2.8∗†	4.5 ± 2.1∗†	3.7 ± 1.6∗†	3.0 ± 1.6†	2.48 ± 1.06†
95% CI	1.4-2.2	6.4-9.4	5.0-8.8	4.4-7.5	3.4-5.7	2.8-4.6	2.1-3.8	1.9-3.1
Range	0.5-3.2	4.7-13.8	3.4-13.1	2.3-11.2	2.3-9.5	2.3-6.6	1.2-6.1	1.1-4.9
Control								
Participant 1	2.8	5.1	4.6	3.5	4.6	2.3	2.7	2.4
Participant 2	1.9	5.6	5.1	3.8	4.1	2.2	2.3	2.5
Participant 3	2.3	8.5	4.2	2.7	2.6	2.2	1.4	1.3
Participant 4	1.1	1.1	1.9	1.5	1.2	1.4	1	0.9
Participant 5	1.3	2.9	2.1	1.9	1.5	1.1	0.9	0.8
Participant 6	1.6	3.8	2.6	1.9	1.7	1.5	1.6	0.9
Participant 7	2	8.5	7.8	5	4	3	3.4	2.8
Participant 8	1.7	4.5	3.6	4	2.8	1.8	1.6	1.9
Participant 9	2	2.8	2.6	2.8	1.6	2.8	1.4	1.4
Participant 10	1.2	5.6	5.3	3	2.4	2	1.9	2.1
Participant 11	1.2	5.2	3.3	3	1.8	1.8	2.1	2.4
Participant 12	1.6	3.6	3.4	2.8	1.4	1.3	2.4	1.8
Participant 13	1.6	5.2	5.3	5.3	3.3	1.8	1.4	1.1
Participant 14	1.9	9.3	7.4	6.8	4.4	3.6	3.4	2.8
Mean ± SD	1.7 ± 0.5	5.3 ± 2.3∗	4.3 ± 1.8∗	3.5 ± 1.4∗	2.7 ± 1.2∗	2.0 ± 0.7∗	1.9 ± 0.8	1.8 ± 0.7
95% CI	1.4-2.0	4.0-6.6	3.3-5.3	2.7-4.2	2.1-3.4	1.7-2.4	1.5-2.4	1.4-2.2
Range	1.1-2.8	1.1-9.3	1.9-7.8	1.5-6.8	1.2-4.6	1.1-3.6	0.9-3.4	0.8-2.8
Glucose, mg ⋅ dL^–1^								
Experimental								
Participant 1	135	93	91	107	125	123	117	101
Participant 2	102	108	112	96	101	108	111	112
Participant 3	103	100	101	98	98	93	100	97
Participant 4	111	104	109	108	112	111	110	110
Participant 5	133	83	88	102	113	115	102	99
Participant 6	88	80	87	90	94	95	101	100
Participant 7	82	94	99	90	89	87	82	79
Participant 8	80	82	85	86	96	95	85	86
Participant 9	106	125	119	102	100	98	106	96
Participant 10	84	99	109	102	96	92	94	89
Participant 11	104	88	96	90	92	96	98	91
Participant 12	120	128	90	91	100	101	100	90
Participant 13	131	96	99	91	99	110	123	132
Participant 14	91	97	99	86	84	82	85	82
Mean ± SD	104.7 ± 18.4	98.2 ± 14.0	98.9 ± 10.0	96.3 ± 7.6	99.9 ± 10.2	100.5 ± 11.1	101.0 ± 11.6	97.6 ± 13.3
95% CI	94.5-114.9	90.5-105.9	93.4-104.4	92.0-100.5	94.3-105.6	94.3-106.6	94.6-107.5	90.2-104.6
Range	80-135	80-128	87-119	86-108	84-125	82-123	82-123	79-132
Control								
Participant 1	94	82	98	93	96	93	90	91
Participant 2	118	112	117	121	113	116	106	109
Participant 3	101	93	93	94	93	94	91	97
Participant 4	117	114	111	119	110	114	110	111
Participant 5	93	97	98	105	105	93	94	94
Participant 6	89	88	94	95	94	93	94	86
Participant 7	116	116	87	84	85	78	85	79
Participant 8	126	98	100	99	102	108	102	100
Participant 9	99	98	91	98	104	98	86	91
Participant 10	124	112	113	121	131	126	110	102
Participant 11	104	86	89	93	99	99	95	101
Participant 12	126	112	94	109	101	102	100	100
Participant 13	97	87	90	95	101	111	112	115
Participant 14	154	107	97	102	127	129	131	125
Mean ± SD	110.9 ± 17.3	99.3 ± 11.9	97.3 ± 9.4	101.1 ± 11.7	103.5 ± 12.6	103.2 ± 13.9	99.9 ± 12.3	99.9 ± 11.7
95% CI	101.4-120.5	92.7-105.9	92.0-102.5	94.6-107.6	96.6-110.5	95.5-110.9	93.1-106.7	93.4-106.3
Range	89-154	82-116	87-117	84-121	85-131	78-129	85-131	86-125

CI, confidence interval; PRE, prior to exercise protocol; SD, standard deviation; T0, immediately after exercise; T20, 20 minutes after exercise; T40, 40 minutes after exercise; T60, 60 minutes after exercise.

∗ Significant increase from PRE.

† Significant session × time interaction at specified time point.

## Discussion

The most important findings of this study were the significant elevations in CD34^+^ cells and platelets above CON values immediately after the EXP exercise session, which could represent another potential mechanism for the noted efficacy of BFR. The results suggest that resistance exercise in men using the Delfi system produces a statistically significant mobilization of HPCs (72% vs 4.3%) and platelets (14% vs 4.9%) to the peripheral circulation, beyond that of the CON session. This finding is consistent with findings in previously published literature showing a general rise in peripheral HPCs after standard non-BFR exercise.^25^^,^^26^^,^^28^^,^^29^ The significant lactate elevation was noted immediately after exercise and from 0 to 40 minutes after the exercise session, which is consistent with previously published findings.^13^, ^14^, ^15^, ^16^, ^17^ This finding shows that the participants were exercising at a high enough level to cause a desired systemic metabolic response.

The higher average platelet count should also be taken into consideration if one wishes to alter the components of a point-of-care blood product.^28^ Previous literature has shown variability in the platelet product yield among commercially available platelet-rich plasma kits.^30^ BFR may be potentially leveraged as a way to noninvasively increase peripheral platelet release prior to blood draw to improve the platelet-rich plasma yield that would be administered. The rise in platelets after the EXP session was consistent with recent findings showing an increase in peripheral mobilization of platelets after vigorous exercise. However, these studies focused on traditional training methods not using BFR.^25^^,^^26^^,^^28^^,^^29^ These results may explain the noted efficacy of BFR versus traditional therapy methods and show that BFR may be leveraged to improve the physiology of the rehabilitating athlete and potentially manipulate point-of-care blood products. Additionally, it is important to consider the individual variability in blood levels, as well as the variability in blood levels at different time points in the same individual.

Lymphocytes and neutrophils were also examined because we hypothesized that these cells could potentially represent indirect markers for the peripheral release of stem cells. There was a significant increase in lymphocyte numbers and, conversely, a significant decrease in average neutrophil numbers immediately after the exercise session. The finding of a significant decrease in average lymphocyte numbers at T60 after the EXP session and a significant increase in neutrophil numbers at both T40 and T60 may represent physiological overcompensation to correct the noted post-exercise changes in an attempt for the body to re-achieve homeostasis. The physiological overcompensation could also explain the significant decrease in average platelet count noted after the EXP session only at T40 after exercise. It is speculated that the significant rise in lymphocyte numbers and converse decrease in neutrophil numbers for the EXP session may represent the release of progenitor cells that were registered as lymphocytes by the automated processing that was used for the CBC analysis.

### Limitations

A limitation of this study was the relatively low number of participants included in each evaluation. This number was due to the selection criteria, as well as the fairly invasive nature of the assessments. The use of manual differentiation of the CBC for post-training blood draws versus our automated processing may also have potentially clarified some of the significant changes noted, specifically the elevation of lymphocytes and, conversely, the significant decrease in average neutrophils. Another limitation of this study was that only male participants were included. The results may differ in female participants.

## Conclusions

Exercise with BFR causes a significant post-exercise increase in peripheral HPCs and platelets, beyond that of standard resistance training.

## Supplementary Data

ICMJE author disclosure forms

## References

[1] Day B. (2018). Personalized blood flow restriction therapy: How, when and where can it accelerate rehabilitation after surgery?. Arthroscopy.

[2] Hylden C., Burns T., Stinner D., Owens J. (2015). Blood flow restriction rehabilitation for extremity weakness: A case series. J Spec Oper Med.

[3] Takarada Y., Takazawa H., Ishii N. (2000). Applications of vascular occlusion diminish disuse atrophy of knee extensor muscles. Med Sci Sports Exerc.

[4] Vechin F.C., Libardi C.A., Conceicao M.S. (2015). Comparisons between low-intensity resistance training with blood flow restriction and high-intensity resistance training on quadriceps muscle mass and strength in elderly. J Strength Cond Res.

[5] Ellefsen S., Hammarstrom D., Strand T.A. (2015). Blood flow-restricted strength training displays high functional and biological efficacy in women: A within-subject comparison with high-load strength training. Am J Physiol Regul Integr Comp Physiol.

[6] Ladlow P., Coppack R.J., Dharm-Datta S. (2018). Low-load resistance training with blood flow restriction improves clinical outcomes in musculoskeletal rehabilitation: A single-blind randomized controlled trial. Front Physiol.

[7] Dankel S.J., Jessee M.B., Abe T., Loenneke J.P. (2016). The effects of blood flow restriction on upper-body musculature located distal and proximal to applied pressure. Sports Med.

[8] Scott B.R., Loenneke J.P., Slattery K.M., Dascombe B.J. (2016). Blood flow restricted exercise for athletes: A review of available evidence. J Sci Med Sport.

[9] Slysz J., Stultz J., Burr J.F. (2016). The efficacy of blood flow restricted exercise: A systematic review & meta-analysis. J Sci Med Sport.

[10] Kim D., Singh H., Loenneke J.P. (2016). Comparative effects of vigorous-intensity and low-intensity blood flow restricted cycle training and detraining on muscle mass, strength, and aerobic capacity. J Strength Cond Res.

[11] Pope Z.K., Willardson J.M., Schoenfeld B.J. (2013). Exercise and blood flow restriction. J Strength Cond Res.

[12] Tennent D.J., Hylden C.M., Johnson A.E., Burns T.C., Wilken J.M., Owens J.G. (2017). Blood flow restriction training after knee arthroscopy: A randomized controlled pilot study. Clin J Sport Med.

[13] Manini T.M., Yarrow J.F., Buford T.W., Clark B.C., Conover C.F., Borst S.E. (2012). Growth hormone responses to acute resistance exercise with vascular restriction in young and old men. Growth Horm IGF Res.

[14] Reeves G.V., Kraemer R.R., Hollander D.B. (2006). Comparison of hormone responses following light resistance exercise with partial vascular occlusion and moderately difficult resistance exercise without occlusion. J Appl Physiol (1985).

[15] Takano H., Morita T., Iida H. (2005). Hemodynamic and hormonal responses to a short-term low-intensity resistance exercise with the reduction of muscle blood flow. Eur J Appl Physiol.

[16] Takarada Y., Nakamura Y., Aruga S., Onda T., Miyazaki S., Ishii N. (2000). Rapid increase in plasma growth hormone after low-intensity resistance exercise with vascular occlusion. J Appl Physiol (1985).

[17] Fujita S., Abe T., Drummond M.J. (2007). Blood flow restriction during low-intensity resistance exercise increases S6K1 phosphorylation and muscle protein synthesis. J Appl Physiol (1985).

[18] Ganesan G., Cotter J.A., Reuland W., Cerussi A.E., Tromberg B.J., Galassetti P. (2015). Effect of blood flow restriction on tissue oxygenation during knee extension. Med Sci Sports Exerc.

[19] Rossi F.E., de Freitas M.C., Zanchi N.E., Lira F.S., Cholewa J.M. (2018). The role of inflammation and immune cells in blood flow restriction training adaptation: A review. Front Physiol.

[20] Nielsen J.L., Aagaard P., Bech R.D. (2012). Proliferation of myogenic stem cells in human skeletal muscle in response to low-load resistance training with blood flow restriction. J Physiol.

[21] Wernbom M., Apro W., Paulsen G., Nilsen T.S., Blomstrand E., Raastad T. (2013). Acute low-load resistance exercise with and without blood flow restriction increased protein signalling and number of satellite cells in human skeletal muscle. Eur J Appl Physiol.

[22] Layne A.S., Larkin-Kaiser K., MacNeil R.G. (2017). Effects of blood-flow restriction on biomarkers of myogenesis in response to resistance exercise. Appl Physiol Nutr Metab.

[23] Ferguson R.A., Hunt J.E.A., Lewis M.P. (2018). The acute angiogenic signalling response to low-load resistance exercise with blood flow restriction. Eur J Sport Sci.

[24] Cesselli D., Beltrami A.P., Rigo S. (2009). Multipotent progenitor cells are present in human peripheral blood. Circ Res.

[25] Marycz K., Mierzejewska K., Smieszek A. (2016). Endurance exercise mobilizes developmentally early stem cells into peripheral blood and increases their number in bone marrow: Implications for tissue regeneration. Stem Cells Int.

[26] Rochefort G.Y., Delorme B., Lopez A. (2006). Multipotential mesenchymal stem cells are mobilized into peripheral blood by hypoxia. Stem Cells.

[27] Liu L., Yu Q., Lin J. (2011). Hypoxia-inducible factor-1alpha is essential for hypoxia-induced mesenchymal stem cell mobilization into the peripheral blood. Stem Cells Dev.

[28] Anz A.W., Parsa R.S., Romero-Creel M.F. (2019). Exercise-mobilized platelet-rich plasma: Short-term exercise increases stem cell and platelet concentrations in platelet-rich plasma. Arthroscopy.

[29] Emmons R., Niemiro G.M., Owolabi O., De Lisio M. (2016). Acute exercise mobilizes hematopoietic stem and progenitor cells and alters the mesenchymal stromal cell secretome. J Appl Physiol (1985).

[30] Fitzpatrick J., Bulsara M.K., McCrory P.R., Richardson M.D., Zheng M.H. (2017). Analysis of platelet-rich plasma extraction: Variations in platelet and blood components between 4 common commercial kits. Orthop J Sports Med.

